# Occurrence and disease burden of respiratory syncytial virus and other respiratory pathogens in adults aged ≥65 years in community: A prospective cohort study in Japan

**DOI:** 10.1111/irv.12928

**Published:** 2021-11-03

**Authors:** Daisuke Kurai, Makiko Natori, Maho Yamada, Richuan Zheng, Yuki Saito, Hiroshi Takahashi

**Affiliations:** ^1^ Department of General Medicine Kyorin University School of Medicine Tokyo Japan; ^2^ Research and Development Division Janssen Pharmaceutical K. K Tokyo Japan; ^3^ Department of Respiratory Medicine Saka General Hospital Miyagi Japan

**Keywords:** acute respiratory disease, community, Japanese older adults, respiratory infection intensity questionnaire (RiiQ™), respiratory pathogens, respiratory syncytial virus

## Abstract

**Background:**

The frequency and clinical profile of respiratory syncytial virus (RSV)–acute respiratory disease (ARD) in older adults in Japan has not been well‐characterized.

**Methods:**

This was a multicenter prospective observational cohort study to evaluate the occurrence rate of ARD in 1000 older adult participants (≥65 years) for 52 weeks during the 2019 to 2020 season. A multiplex polymerase chain reaction panel was used for pathogen detection in nasopharyngeal swab from participants diagnosed with ARD. Symptoms and impact of ARD was assessed using the Respiratory Infection Intensity and Impact Questionnaire (RiiQ™). The study was registered at UMIN (https://www.umin.ac.jp/ctr/): UMIN000037891.

**Results:**

RSV–ARD was detected in 24/1000 (2.4%) participants and RSV‐lower respiratory tract disease in 8/1000 (0.8%) participants. The median duration of RSV–ARD was 18 days. All 24 participants had utilized the medical services of outpatient visits and only 1 (4.2%) participant was hospitalized for RSV–ARD. The most common viruses other than RSV that caused ARD (detected in >10 participants) were human rhinovirus/enterovirus, parainfluenza 3, coronavirus OC43, human metapneumovirus, and influenza A/H1. The most frequent symptoms of RSV–ARD were cough, sore throat, nasal congestion, and expectoration.

**Conclusions:**

RSV was reported as a major pathogen for respiratory infections in older adults in Japan.

## 1 INTRODUCTION

Respiratory syncytial virus (RSV) is a common pathogen responsible for mild cold‐like symptoms to lower respiratory tract infection (LRTI). Generally people recover within 2 weeks, but it can be serious in infants, children <5 years, older adults (especially ≥60 years), immunocompromised, or people with underlying cardio‐pulmonary conditions.^1^ Globally, in 2015, approximately 33.1 million children <5 years reported RSV‐associated acute LRTI, of which 3.2 million were hospitalized and 59,600 died, while 336,000 older adults were hospitalized due to RSV‐associated acute respiratory infection, of which 14,000 died.^2^, ^3^ Annually, over 177,000 older adults are hospitalized and 14,000 die in the United States (US) with RSV infection.^1^

Older adults have higher risk of severe complications from RSV due to immunosenescence.^1^ A 4‐year prospective study in the US during 1999 to 2003 showed that 3%–7% of healthy older adults, 4%–10% of high‐risk patients, and 8%–13% of hospitalized patients had RSV infection.^4^ Another prospective observational cohort study in Europe (EU) during 2018 to 2019 showed that RSV infection is prevalent in community‐dwelling older adults (≥60 years) with an annual incidence of 2.1%–4.9%; however, it rarely causes severe disease.^5^ In long‐term care facilities, RSV is estimated to infect 5%–10% of residents per year with significant rates of pneumonia (10%–20%) and death (2%–5%).^6^

In the EU and the US, RSV is increasingly recognized, including impact on older adults; however, little is known about RSV infection in Japan.^7^ Several prophylactic vaccines against RSV disease in older adult populations are currently under development. As Japan has a significant population over 60 years of age (~35.6 million),^8^ it is important to understand the burden of RSV disease in this population.

The objectives of this study were to estimate the occurrence rate of RSV infection, and to characterize the RSV disease outcome in older adults in Japan.

## 2 METHODS

### 2.1 Study population

Ambulatory adults (≥65 years) living in Japan in community or assisted‐living or long‐term care residential facilities, and who were, healthy or with clinically stable medical conditions, were enrolled. Participants with acute respiratory disease (ARD) at consent and Week 0 (baseline), or who had received experimental antiviral drugs or vaccines for RSV within 6 months prior to the study, or had a life expectancy <1 year, were excluded.

### 2.2 Study design

**FIGURE 1:**
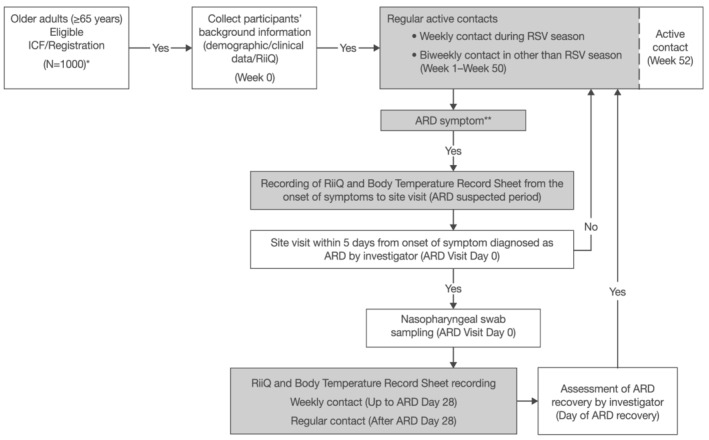
Schematic diagram of the study. Greyed cells in the above figure indicate scheduled activities to be performed at home. White cells in the above figure indicate scheduled activities to be performed at the study site. *Of which >30% aged 65 to 74 years and >30% aged ≥75 years and older (including >5% of 85 years and older as possible). **In the case that participants recovered and symptoms recurred, ARD was defined as a single episode or a separate episode of respiratory illness by the clinical judgment of the investigator; however, the episodes of respiratory illness that occurred >28 days of each other as a separate episode of illness were to be counted.^10^ Abbreviations: ARD = acute respiratory disease, ICF = informed consent form, RiiQ™ = Respiratory Infection Intensity Questionnaire, RSV = respiratory syncytial virus

The study was conducted at 10 sites in Japan from April 2019 to July 2020. Participants were followed up bi‐weekly (weekly during RSV season, predefined from 01 June 2019 to 30 November 2019)^9^ through telephone to monitor ARD. Each participant was observed for 52 weeks from the day of registration or until withdrawal (Figure 1).

The study was conducted in accordance with the principles defined in the Declaration of Helsinki, Ethical Guidelines for Medical and Health Research Involving Human Subjects, Japanese regulations, and sponsor policy.

### 2.3 Data collection

Clinical data of coexisting medical conditions including conditions conferring increased risk for severe RSV disease: congestive heart failure, coronary artery disease, chronic obstructive pulmonary disease, asthma, or immunocompromised condition due to medications, transplant, asplenia, or innate immunodeficiency, etc., were collected at Week 0. The participants visited study sites after the onset of any ARD symptoms, and a nasopharyngeal swab sample per episode was collected if they were diagnosed with ARD by the investigator. The ARD symptoms to be filled in the survey sheet included LRTI (cough, shortness of breath, sputum production, and wheezing); upper respiratory tract infection (URTI; nasal congestion and sore throat); and systemic symptoms (headache, fatigue, fever [>38°C], feverishness, and myalgia) by the investigator. The Respiratory Panel of BIOFIRE® FILMARRAY® Multiplex polymerase chain reaction (PCR) system was used for the identification of pathogens in the nasopharyngeal swabs. The RSV‐positive samples were further analyzed for RSV A or B subtyping, performed using RealStar® RSV reverse transcriptase (RT)‐PCR kit 3.0. The Respiratory Infection Intensity and Impact Questionnaire (RiiQ™) was used to assess ARD symptoms by the participants. Its dimensions included LRTI, URTI, and systemic symptoms. The RiiQ™, adapted from Influenza Intensity and Impact Questionnaire (Flu‐iiQ™), was used under license from Measured Solutions for Health P/L, Australia. This is a patient‐reported outcome instrument designed to assess symptom intensity and impact of respiratory infection. The RiiQ™ scales are scored as an average of each symptom related to each scale. Each symptom was scored from 0 (none) to 3 (severe).^11^, ^12^ The RiiQ™ and body temperature were assessed daily in all ARD participants from the onset of ARD symptoms until recovery as confirmed by investigators or until Week 52 (if participants did not recover). Investigator also collected information regarding utilization of medical services (outpatient visits, emergency room [ER] visits, hospitalizations, or intensive care unit [ICU] hospitalizations) during the ARD period and assessed the impact of ARD on basic functioning and independence status using the Barthel Index of Activities of Daily Living (BIADL) Assessment Sheet. The BIADL is a 10‐item ordinal scale with scores ranging from 0 to 20 (lower scores indicating increased disability).^13^ Functional status >16 was defined as independent.^14^, ^15^

### 2.4 Statistical Methods

#### 2.4.1 Sample size determination

Approximately 1000 participants (>30% aged 65 to 74 years and >30% aged ≥75 years [including >5% aged ≥85 years]) were planned. The occurrence rate of RSV infection in older adult population was conservatively estimated to be 2%.^4^ A total of 1000 participants were expected to allow for precision of estimation of the true attack rate of 2%, as measured by the width of the two‐sided 95% confidence interval (CI).

#### 2.4.2 Endpoints

##### Primary

Occurrence of RSV–ARD (defined as new onset or worsening of ≥1 symptom of URTI and/or LRTI with or without systemic symptoms with an RT‐PCR confirmed diagnosis of RSV) in older adults in Japan during the study period.

##### Secondary

Occurrence of RSV‐lower respiratory tract disease (LRTD; defined as new onset or worsening of at least 1 symptom of LRTI, assessed by the investigator, with an RT‐PCR confirmed diagnosis of RSV), occurrence of ARD/LRTD caused by respiratory pathogens other than RSV; outcome of RSV–ARD and ARD caused by respiratory pathogens other than RSV (recovery conditions, signs and symptoms, types of medical services utilized for ARD); and occurrence rates of RSV–ARD or ARD caused by other respiratory pathogens by subgroups (age groups, gender, presence of underlying comorbidities or immunosuppression, functional status, smoking history, influenza vaccination in 2019/2020, and place of residence).

Of the above secondary endpoints, occurrence of LRTD caused by other respiratory pathogens, occurrence rate of RSV–ARD by smoking history and influenza vaccination in 2019/2020, and occurrence of ARD caused by other respiratory pathogens by demographic data were evaluated as post hoc analyses.

##### Exploratory

Number of RT‐PCR‐confirmed RSV–LRTD cases in clinical case definitions, defined as ≥3 LRTI symptoms for case definition #1, ≥2 LRTI symptoms for case definition #2 and ≥2 LRTI symptoms or ≥1 LRTI combined with ≥1 URTI and ≥1 systemic symptom for case definition #3, based on clinical outcome assessed by RiiQ™ and Body Temperature Record Sheet; trend of the seasonality of RT‐PCR‐confirmed RSV infection and other respiratory pathogens; and change from baseline functioning and independence status assessed by clinician using BIADL questionnaire.

The diagnosis and severity at ARD diagnosis by investigators, and the time from the onset day of ARD symptoms to the day of ARD diagnosis (ARD Visit Day 0) were also evaluated as post hoc analyses.

#### 2.4.3 Statistical analyses

This was an exploratory descriptive study; therefore, no hypothesis testing was conducted. The precision of the estimate of the occurrence rate was determined using the Clopper–Pearson exact 95% CI. All participants having baseline (Week 0) data were included in the analyses. All data were summarized descriptively.

## 3 RESULTS

### 3.1 Study population

A total of 1000 participants were enrolled; 913 (91.3%) completed and 87 (8.7%) discontinued the study. Discontinuations were due to consent withdrawal (*n* = 58 [5.8%]), lost to follow‐up (*n* = 22 [2.2%]), death (*n* = 6 [0.6%]), and failure to comply with protocol requirements (*n* = 1 [0.1%]). Overall, 313/1000 (31.3%) participants had 459 ARD visits (Table 1). Overall compliance rate of recording patient reported outcome during each ARD period was 81.8% (data not shown).

**TABLE 1:** Summary of participant disposition, demographic variables, and baseline characteristics

Variables	Value
Participants enrolled	1000
Completed study n (%)	913 (91.3)
Discontinued from the study n (%)	87 (8.7)
Reason for discontinuation	
Lost to follow‐up	22 (2.2)
Consent withdrawal	58 (5.8)
Death	6 (0.6)
Repeated failure to comply with protocol requirements	1 (0.1)
Time of discontinuation *n* (%)	
Before start of RSV season	32 (3.2)
During RSV season	16 (1.6)
After RSV season	39 (3.9)
Number of ARD visits	459
Participants with ARD visit *n* (%)	313 (31.3)
Sex *n* (%)	
Female	551 (55.1)
Male	449 (44.9)
Age (years)	
Mean (SD)	74.3 (6.23)
Age group (years) *n* (%)	
65–74	543 (54.3)
75–84	383 (38.3)
≥85	74 (7.4%)
Resident information *n* (%)	
Home	996 (99.6%)
Assisted‐living or long‐term care residential facility	4 (0.4%)
Coexisting medical condition *n* (%)	
No	42 (4.2%)
Yes	958 (95.8%)
Chronic heart disease	
Congestive heart failure	18 (1.8%)
Coronary artery disease	66 (6.6%)
Chronic lung disease	
COPD	31 (3.1%)
Asthma	104 (10.4%)
Immunocompromised	7 (0.7%)
Other	923 (92.3%)

Abbreviation: ARD = acute respiratory disease, COPD = chronic obstructive pulmonary disease, *n* = number of participants, RSV = respiratory syncytial virus, SD = standard deviation.

The proportion of females (55.1%) was higher than males (44.9%); the mean (standard deviation [SD]) age was 74.3 (6.23) years; more than half of participants (54.3%) were 65 to 74 years. Most participants were home residents (99.6%), living with family/cohabiter (80.9%), nonsmokers (57.6%), and were vaccinated with influenza vaccine in 2019/2020 (64.5%). At baseline, most participants (998 [99.8%]) had a functional status of >16. The most common (reported in >10% of participants) coexisting medical condition was asthma (*n* = 104 [10.4%]) among conditions with increased risk for severe RSV (Tables 1 and 2).

**TABLE 2:** Summary of occurrence rate of RSV–ARD or ARD caused by influenza A/H1 by demographic and baseline characteristics subgroups during the whole study period

Subgroups	*N* = 1000	RSV–ARD		ARD by influenza A/H1	
		*n* (%)	95% CI	*n* (%)	95% CI
		24 (2.4)	1.54, 3.55	11 (1.1)	0.55, 1.96
Age (years)					
65–74	543	14 (2.6)	1.42, 4.29	8 (1.5)	0.64, 2.88
75–84	383	8 (2.1)	0.91, 4.07	3 (0.8)	0.16, 2.27
≥ 85	74	2 (2.7)	0.33, 9.42	0	—
Sex					
Female	551	19 (3.4)	2.09, 5.33	8 (1.5)	0.63, 2.84
Male	449	5 (1.1)	0.36, 2.58	3 (0.7)	0.14, 1.94
Presence of underlying comorbidities or immunosuppression					
Yes	958	23 (2.4)	1.53, 3.58	11 (1.1)	0.57, 2.05
Chronic heart disease (CHF + CAD)	78	1 (1.3)	0.03, 6.94	1 (1.3)	0.03, 6.94
Chronic lung disease (COPD+Asthma)	127	4 (3.1)	0.86, 7.87	2 (1.6)	0.19, 5.57
Immunocompromised	7	0	—	0	—
Other	923	23 (2.5)	1.59, 3.72	11 (1.2)	0.60, 2.12
Diabetes mellitus (SMQ)	424	9 (2.1)	0.98, 3.99	4 (0.9)	0.26, 2.40
Hematological malignant tumors (SMQ)	2	0	—	0	—
No	42	1 (2.4)	0.06, 12.57	0	—
Functional status^a^					
≤16	2	0	—	0	—
>16	998	24 (2.4)	1.55, 3.56	11 (1.1)	0.55, 1.96
Place of residence					
Home	996	24 (2.4)	1.55, 3.56	11 (1.1)	0.55, 1.97
Assisted‐living or long‐term care residential facility	4	0	—	0	—
Structure of family living together/cohabiters					
Alone	191	5 (2.6)	0.86, 6.00	0	—
Living with family/cohabiter	809	19 (2.3)	1.42, 3.64	11 (1.4)	0.68, 2.42
Smoking history					
Smoker	85	1 (1.2)	0.03, 6.38	0	—
Former smoker	339	8 (2.4)	1.02, 4.60	2 (0.6)	0.07, 2.11
Nonsmoker	576	15 (2.6)	1.46, 4.26	9 (1.6)	0.72, 2.95
Seasonal influenza vaccination					
Yes	645	15 (2.3)	1.31, 3.81	8 (1.2)	0.54, 2.43
No	330	8 (2.4)	1.05, 4.72	2 (0.6)	0.07, 2.17
Unknown or missing	25	1 (4.0)	0.10, 20.35	1 (4.0)	0.10, 20.35

Abbreviations: ARD = acute respiratory disease, CAD = coronary artery disease, CHF = congestive heart failure, CI = confidence interval, COPD = chronic obstructive pulmonary disease, RSV = respiratory syncytial virus, SMQ = Standardised MedDRA Queries.

^a^ The score is from the Barthel Index of ADL.

### 3.2 Primary outcomes

RSV–ARD was detected in 24/1000 (2.4%) participants (95% CI: 1.54, 3.55) by RT‐PCR; of which, 13/24 (54.2%) and 11/24 (45.8%) had Subtypes A and B RSV–ARD, respectively (Table 3).

**TABLE 3:** Summary of occurrence rate of RSV–ARD, RSV–LRTD, and ARD and LRTD by other respiratory pathogens during the whole study period

	RSV–ARD (*N* = 1000)			RSV–LRTD (*N* = 1000)		
	Total	Subtype A	Subtype B	Total	Subtype A	Subtype B
*n* (%)	24 (2.4%)	13 (1.3%)	11 (1.1%)	8 (0.8%)	7 (0.7%)	1 (0.1%)
95% CI	1.54, 3.55	0.69, 2.21	0.55, 1.96	0.35, 1.57	0.28, 1.44	0.00, 0.56

Pathogens	ARD by other pathogens			LRTD by other pathogens		
	*n* (%)	95% CI		*n* (%)	95% CI	
Coronavirus 229E	3 (0.3)	0.06, 0.87		—	—	
Coronavirus HKU1	1 (0.1)	0.00, 0.56		—	—	
Coronavirus OC43	12 (1.2)	0.62, 2.09		2 (0.2)	0.02, 0.72	
Coronavirus NL63	4 (0.4)	0.11, 1.02		—	—	
Human Metapneumovirus	12 (1.2)	0.62, 2.09		6 (0.6)	0.22, 0.72	
Human Rhinovirus/Enterovirus	100 (10.0)	8.21, 12.03		21 (2.1)	1.30, 3.19	
Influenza A/H1	11 (1.1)	0.55, 1.96		2 (0.2)	0.02, 0.72	
Influenza B	1 (0.1)	0.00, 0.56		—	—	
Parainfluenza 1	2 (0.2)	0.02, 0.72		—	—	
Parainfluenza 2	3 (0.3)	0.06, 0.87		1 (0.1)	0.00, 0.56	
Parainfluenza 3	20 (2.0)	1.23, 3.07		3 (0.3)	0.06, 0.87	
Parainfluenza 4	1 (0.1)	0.00, 0.56		—	—	
Bordetella pertussis	1 (0.1)	0.00, 0.56		—	—	
Mycoplasma pneumoniae	1 (0.1)	0.00, 0.56		—	—	

Abbreviations: ARD = acute respiratory disease, CI = confidence interval, LRTD = lower respiratory tract disease, RSV = respiratory syncytial virus.

Note: LRTD cases are shown in this table as ARD.

#### 3.2.1 Secondary outcomes

RSV–LRTD, as assessed by the investigator was detected in 8/1000 (0.8%) participants (95% CI: 0.35, 1.57); 7 (0.7%) and 1 (0.1%) had Subtypes A and B RSV–LRTD, respectively (Table 3).

The median (range) duration of RSV–ARD was 18.0 (10 to 33) days (Table S1). The most frequent symptoms recorded by the participants (mean change of >1.0 in symptom score from baseline) for RSV–ARD were cough, sore throat, nasal congestion, and expectoration. For most participants (*n* = 15), symptoms improved by Day 14; however, for few participants (*n* = 7), the symptoms lasted for 21 to 28 days after onset (data not shown). Respiratory tract symptoms tended to take longer to return to baseline for RSV and human metapneumovirus than for influenza A/H1 (Figure S1). The body temperature of participants with the RSV–ARD was stable over time (Days 0 to 28; 35.0°C to 38.4°C) and was similar in Subtypes A and B. Only 1 participant with Subtype B had fever (38.4°C) on Day 4 from onset of ARD. All 24 participants detected with RSV had utilized medical services of outpatient visits and 1 (4.2%) was hospitalized for 10 days for RSV–ARD. No participant died, was admitted to the ICU, or visited an ER due to RSV–ARD.

Pathogens were detected in 199/459 samples. Coinfection was confirmed in 2 samples, and others detected a single pathogen. The most common viruses other than RSV that caused ARD (detected in >10 participants) were human rhinovirus/enterovirus (100 [10.0%]), parainfluenza 3 (20 [2.0%]), coronavirus OC43 and human metapneumovirus (12 [1.2%]), and influenza A/H1 (11 [1.1%]). ARD due to influenza B virus was reported in 1 participant. Only human rhinovirus/enterovirus‐infected participants reported with >1 episode of ARD. The most common viruses other than RSV that caused LRTD (detected in >10 participants) was human rhinovirus/enterovirus (21/1000 [2.1%]) (Table 3).

No participant died or was admitted to the ICU due to ARD caused by pathogens other than RSV. The median (range) duration of ARD caused by those viruses or bacteria (detected in >10 participants) was 20.5 (10–36) days, 15.5 (9–31) days, 15.0 (5–55) days, 15.0 (5–38) days, and 12.5 (6–35) days for human metapneumovirus, parainfluenza 3, human rhinovirus/enterovirus, influenza A/H1, and coronavirus OC43, respectively (Table S1). The most frequent symptoms recorded by participants were cough, sore throat, expectoration, and loss of appetite with parainfluenza 3; cough, feeling feverish, and fatigue with influenza A/H1; cough and sore throat with human rhinovirus/enterovirus; cough and expectoration with human metopneumovirus; and nasal congestion with coronavirus OC43. Fever was reported with human metapneumovirus, human rhinovirus/enterovirus, influenza A/H1, parainfluenza 2, parainfluenza 3, and *Bordetella pertussis* (data not shown). Two participants with ARD were hospitalized, 1 due to human metapneumovirus for 6 days and 1 due to influenza A/H1 for 9 days.

Of the 24 participants who had RSV–ARD; 14 (58.3%) were 65‐ to 74‐years‐old, 8 (33.3%) were 75‐ to 84‐years‐old, and 2 (8.3%) were ≥85‐years‐old. The occurrence rate of RSV–ARD was higher in females (19/551; 3.4%) than males (5/449; 1.1%). Four of 127 (3.1%), 9/424 (2.1%), and 1/78 (1.3%) participants with underlying comorbidities of chronic lung disease, diabetes mellitus, and chronic heart disease, respectively, had RSV–ARD. RSV–ARD was detected in 15/576 (2.6%) nonsmokers, 8/339 (2.4%) former smokers and 1/85 (1.2%) smokers with a history of smoking; and in 15/645 (2.3%) vaccinated and 8/330 (2.4%) not vaccinated among seasonal influenza vaccination (Table 2).

Of the other respiratory pathogens, only influenza A was analyzed for subgroups, given influenza A is a well‐recognized respiratory disease caused ARD. All 11 participants with influenza A detected influenza A/H1. Of the 11 participants; 8 (72.7%) were 65‐ to 74‐years‐old and 3 (27.3%) were 75‐ to 84‐years‐old. The occurrence rate of ARD by influenza A/H1 was higher in females (8/551; 1.5%) than males (3/449; 0.7%). Two of 127 (1.6%), 1/78 (1.3%), and 4/424 (0.9%) participants had ARD by influenza A/H1 with underlying comorbidities of chronic lung disease, chronic heart disease, and diabetes mellitus, respectively. The ARD by influenza A/H1 was detected in 9/576 (1.6%) nonsmokers and 2/339 (0.6%) former smokers among smoking history; and in 8/645 (1.2%) participants vaccinated and 2/330 (0.6%) not vaccinated among seasonal influenza vaccination (Table 2).

#### 3.2.2 Exploratory outcome

The three case definitions ranged from the most stringent case definition #1 to the least stringent case definition #3. Of the 1000 participants, 17 (1.7%), 22 (2.2%), and 23 (2.3%) participants had RT‐PCR confirmed RSV–LRTD based on case definitions #1, #2, and #3, respectively. One participant was not classified in any case definition (Table 4).

**TABLE 4:** Summary of RSV participants with clinical case definitions #1 to #3 during the whole study period

Clinical case definitions	Total		Subtype A		Subtype B	
	*n* (%)	95% CI	*n* (%)	95% CI	*n* (%)	95% CI
#1^a^	17 (1.7)	0.99, 2.71	10 (1.0)	0.48, 1.83	7 (0.7)	0.28, 1.44
#2^b^	22 (2.2)	1.38, 3.31	13 (1.3)	0.69, 2.21	9 (0.9)	0.41, 1.70
#3^c^	23 (2.3)	1.46, 3.43	13 (1.3)	0.69, 2.21	10 (1.0)	0.48, 1.83
No	1 (0.1)	0.00, 0.56	0	—	1 (0.1)	0.00, 0.56

Abbreviations: CI = Confidence Interval, LRTI = lower respiratory tract infection, RSV = Respiratory Syncytial Virus.

^a^ ≥3 symptoms of LRTI (new onset or worsening).

^b^ ≥2 symptoms of LRTI (new onset or worsening).

^c^ ≥2 symptoms of LRTI OR ≥1 LRTI combined with ≥1 symptom of URTI and ≥1 systemic symptom (new onset or worsening).

**FIGURE 2:**
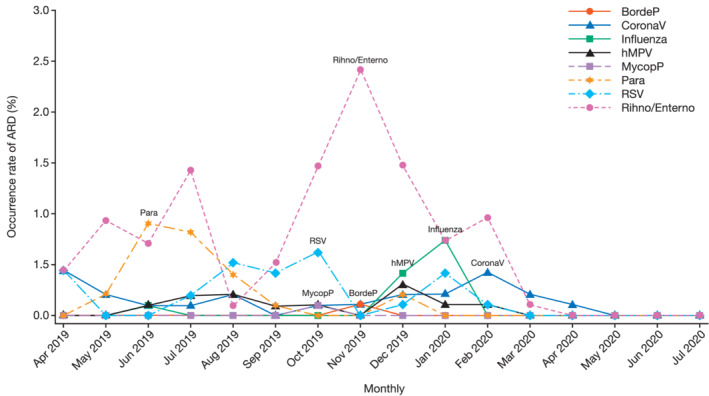
Seasonality of occurrence of acute respiratory disease (ARD) by each pathogen during the whole study period. Abbreviations: BordeP = *Bordetella Pertussis* , CoronaV = Coronavirus, hMPV = Human Metapneumovirus, MycopP = *Mycoplasma Pneumoniae* , Para = Parainfluenza, Rhino/Entero = Human Rhinovirus/Enterovirus, RSV = Respiratory Syncytial Virus

Of the 24 RSV–ARD cases, 17 were reported during the predefined RSV season (01 June 2019 to 30 November 2019); 1 reported before the RSV season and 6 reported after the RSV season. The occurrence of RSV–ARD ranged between 0.4% to 0.6% from August 2019 to October 2019, and in January 2020. The occurrence of ARD (detected in >10 participants) due to pathogens was highest as follows: parainfluenza, RSV, human rhinovirus/enterovirus, human metapneumovirus, influenza, and coronavirus in June 2019, October 2019, November 2019, December 2019, January 2020, and February 2020, respectively (Figure 2).

No changes from baseline in the BIADL were observed during ARD period.

Of 459 ARD episodes, 379 were URTI and 80 were LRTI. The majority of RSV–ARD were mild (21/24 [87.5%]); 3 (12.5%) moderate episodes of ARD were reported. The majority of ARDs due to influenza A/H1 and human metapneumovirus were mild (8/11 [72.7%] and 10/12 [83.3%] participants, respectively), and 3/11 (27.3%) episodes in influenza A/H1 and 2/12 (16.7%) episodes in human metapneumovirus were reported as moderate or severe. More than 93% episodes in human rhinovirus/enterovirus, while 100% episodes in coronavirus OC43 and parainfluenza 3 episodes were mild in severity (Table 5). Based on symptoms recorded by the investigator at ARD diagnosis, cough was commonly observed with moderate or severe intensity and no specific differences in symptoms were identified among pathogens. Wheezing was the most frequent symptom in RSV compared to other pathogens, and moderate to severe upper respiratory symptoms (nasal congestion and sore throat) were observed in RSV. Moderate or severe systemic symptoms, particularly, fever, feverishness, and myalgia, were observed in influenza as well‐recognized, whereas only 1 subject with severe feverishness was reported in RSV (Table S2). The median duration (range) from the onset day of ARD symptoms to the day of ARD diagnosis (ARD Visit Day 0) in RSV was relatively longer (2.0 [0 to 9] days) than influenza A/H1 (1.0 [0 to 7] days) and comparable with other respiratory pathogens (2.0 [1 to 9] days for human metapneumovirus, 2.0 [0 to 3] days for parainfluenza 3, 2.0 [0 to 10] days for human rhinovirus/enterovirus and 2.5 [1 to 5] days for coronavirus OC43).

**TABLE 5:** Summary of diagnosis of ARD by investigators (pathogens detected in >10 samples)

All ARD and ARD with pathogen detected	All	RSV	Influenza A/H1	Human Metapneumovirus	Human rhinovirus/Enterovirus	Coronavirus OC43	Parainfluenza 3
	*N* = 459 *n*%	*N* = 24 *n*%	*N* = 11 *n*%	*N* = 12 *n*%	*N* = 105 *n*%	*N* = 12 *n*%	*N* = 20 *n*%
Diagnosis of ARD							
LRTI	80 (17.4)	8 (33.3)	2 (18.2)	6 (50.0)	21 (20.0)	2 (16.7)	3 (15.0)
URTI	379 (82.6)	16 (66.7)	9 (81.8)	6 (50.0)	84 (80.0)	10 (83.3)	17 (85.0)
Mild	436 (95.0)	21 (87.5)	8 (72.7)	10 (83.3)	98 (93.3)	12 (100.0)	20 (100.0)
Moderate	22 (4.8)	3 (12.5)	2 (18.2)	2 (16.7)	7 (6.7%)	0	0
Severe	1 (0.2)	0	1 (9.1)	0	0	0	0

*Note*: The severity was assessed by the investigator.

Abbreviations: ARD = acute respiratory disease, LRTI = lower respiratory tract infection, *N* = total number of ARD episodes, *n* = number of ARD episodes in each category, RSV = respiratory syncytial virus, URTI = upper respiratory tract infection.

## 4 DISCUSSION

This was the first prospective observational study to identify the occurrence rate of RSV infection in older adults (aged ≥65 years) in the Japanese community. This study characterized the disease outcome of older adults in Japan with RSV associated ARD by following their disease, by recording RiiO™ and body temperature from onset of ARD symptoms until resolution or through 52 weeks.

RSV was identified as a major respiratory pathogen and represented a comparable burden with other major pathogens such as influenza A/H1 and human metapneumovirus in the older population in Japan (2.4%, 1.1%, and 1.2% of participants, respectively). The occurrence rate of RSV–ARD confirmed by RT‐PCR in older adults in Japan identified in this study was comparable with the older adults of US and Europe (2.4% vs. 2.1% to 7%).^4^, ^5^ Most of the RSV–ARD cases occurred during RSV season in Japan.^9^

One participant each with RSV, Influenza A/H1, and human metapneumovirus infection required hospitalization. There were no ARD‐related deaths either due to RSV or other respiratory pathogens. Similar number of participants reported moderate/severe episodes of ARD in participants infected with RSV, influenza A/H1, and human metapneumovirus. Most participants had received influenza vaccination. The median (range) duration of ARD was 20.5 (10 to 36) days for human metapneumovirus, 18.0 (10 to 33) days for RSV, 15.5 (9 to 31) days for parainfluenza 3, 15.0 (5 to 55) days for human rhinovirus/enterovirus, 15.0 (5 to 38]) days for influenza A/H1, and 12.5 (6 to 35) days for coronavirus OC43. The duration of RSV–ARD observed in this study was consistent with other studies conducted in older adults in the US and EU.^4^, ^5^ Overall, these data suggest that RSV, human metapneumovirus and influenza A/H1 have similar disease burden, but additional data are needed to firmly conclude.

Given that the symptom‐profile makes it difficult to clinically distinguish between RSV and other respiratory viruses, a laboratory‐based viral testing is necessary. However, in this study, it was found that the frequency of ARD symptoms and their course differed among viruses. RSV, like human metapneumovirus, tended to have prolonged lower respiratory tract symptoms, which was consistent with the literature.^16^, ^17^ For influenza A/H1, systemic symptoms, such as fever in the early stages of the disease was prominent. The rate of LRTI was 50% for human metapneumovirus, 33.3% for RSV, and 15% to 20% for other pathogens.

The occurrence rate of ARD due to RSV was 3.4% (95% CI: 2.09, 5.33) for females and 1.1% (95% CI: 0.36, 2.58) for males. Similarly, the occurrence rate of ARD due to influenza A/H1 was 1.5% for females and 0.7% for males. Results confirm that chronic lung disease is one of the risk factors for RSV–ARD,^18^, ^19^, ^20^ whereas there were no differences among the risk factors in the occurrence rate of ARD due to influenza A/H1. The small number of participants identified with RSV–ARD in each subgroup makes it difficult to interpret the differences among subgroups of smoking history and seasonal influenza vaccination. The occurrence rate of RSV–ARD was highest during August 2019 and October 2019, which was consistent with the RSV season in Japan.^9^ The occurrence rate of RSV–ARD was also high in January 2020.

No impact of ARD was observed on the functioning and independence of participants based on BIADL disability scores when comparing baseline to end of season. Since participants were encouraged to visit sites within 5 days from onset of any ARD symptoms during the study, it was difficult to definitively characterize ARD symptoms for RSV at the time of ARD diagnosis. Participants with influenza reported systemic symptoms, visited the sites 1 day earlier in time to ARD diagnosis from ARD onset, than other pathogens including RSV reported upper or lower respiratory symptoms.

Severe morbidity and mortality were seen in older adults hospitalized with RSV infection in an earlier study conducted in the US.^21^ Another study conducted in EU confirmed that RSV is prevalent in community‐dwelling older adults.^5^ Hence, there is a need for vaccine development against RSV disease for the older adult populations. The results from the current study helped to estimate the occurrence rate of RSV infection and to characterize the RSV disease outcome in older adults in Japan.

This study has following limitations: the study was designed for only 1 RSV season in 2019 with 1000 participants, resulting that the relatively low numbers of cases reported in each pathogen make it difficult to compare disease characteristics among subgroups or pathogens; the impact of COVID‐19 pandemic occurred in March 2020 was unknown on the occurrence of ARD by respiratory pathogens and the study site visits; there could be an underestimation of severe ARD cases requiring hospitalization given that 90% of participants were enrolled from general practitioner sites and a participant with severe symptoms could visit a hospital site other than study sites and such case was not recorded in the study.

## 5 CONCLUSIONS

RSV was reported as a major pathogen causing respiratory infection and represents a burden as significant as influenza in elderly Japanese community setting. The evidence of medical needs for development of vaccine to prevent RSV infection was generated.

## CONFLICT OF INTEREST

Potential conflict of interest: NM, YM, ZR, and SY are full time employees and KD had been a consultant of Janssen Pharmaceutical K.K., Tokyo, Japan. NM and YM are J&J stockholders.

No conflict of interest: TH reports no conflicts of interest in this work.

## AUTHOR CONTRIBUTIONS

**Daisuke Kurai:** Conceptualization; methodology; writing‐review editing; supervision. **Makiko Natori:** Conceptualization; methodology; project administration; writing‐review editing. **Maho Yamada:** Conceptualization; writing‐review editing; project administration. **Richuan Zheng:** Formal analysis; methodology; writing‐review editing. **Yuki Saito:** Formal analysis; methodology; writing‐review editing. **Hiroshi Takahashi:** Conceptualization; writing‐review editing; investigation; resources; supervision.

## ETHICS APPROVAL STATEMENT

The study was approved by the Saka General Hospital ethics committee (#18–03‐49), and by the ethics committee at each study site.

## PATIENT CONSENT STATEMENT

Informed consent was obtained from all participants prior to enrolment.

## PERMISSION TO REPRODUCE MATERIAL FROM OTHER SOURCES

No materials were reproduced from other sources.

### PEER REVIEW

The peer review history for this article is available at https://publons.com/publon/10.1111/irv.12928.

## Supporting information


**Table S1:** Summary of Duration of ARD Caused by RSV and Different Types of Respiratory Pathogens Other Than RSV

**Table S2:** Summary of Symptom and Severity at ARD Diagnosis by Investigators (Pathogens Detected in >10 Samples)

**Data S1.** Supporting information

## Data Availability

The data that support the findings of this study are available on request from the corresponding author. The data are not publicly available due to privacy or ethical restrictions.
