# Supplementary material for: Occurrence and disease burden of respiratory syncytial virus and other respiratory pathogens in adults aged ≥65 years in community: A prospective cohort study in Japan
Source: Influenza Other Respir Viruses. 2021 Nov 3;16(2):298–307. doi: 10.1111/irv.12928 (PMC8818832; doi:10.1111/irv.12928)
Supplement: Supplementary file 1 — Table S1: Summary of Duration of ARD Caused by RSV and Different Types of Respiratory Pathogens Other Than RSV [file IRV-16-298-s001.docx]

**Table 1_SuppInfo: Summary of Duration of ARD Caused by RSV and Different Types of Respiratory Pathogens Other Than RSV**

| **Pathogens** | **Duration of ARD (days)** | | | |
| --- | --- | --- | --- | --- |
|  | **N=1000**  **n (%)** | **Minimum** | **Median** | **Maximum** |
| RSV | 24 (2.4) | 10 | 18.0 | 33 |
| Coronavirus 229E | 3 (0.3) | 19 | 29.0 | 33 |
| Coronavirus HKU1 | 1 (0.1) | 18 | 18.0 | 18 |
| Coronavirus OC43 | 12 (1.2) | 6 | 12.5 | 35 |
| Coronavirus NL63 | 4 (0.4) | 7 | 18.0 | 32 |
| Human Metapneumovirus | 12 (1.2) | 10 | 20.5 | 36 |
| Human Rhinovirus / Enterovirus | 100 (10.0) | 5 | 15.0 | 55 |
| Influenza A/H1 | 11 (1.1) | 5 | 15.0 | 38 |
| Influenza B | 1 (0.1) | 17 | 17.0 | 17 |
| Parainfluenza 1 | 2 (0.2) | 17 | 36.5 | 56 |
| Parainfluenza 2 | 3 (0.3) | 6 | 9.0 | 10 |
| Parainfluenza 3 | 20 (2.0) | 9 | 15.5 | 31 |
| Parainfluenza 4 | 1 (0.1) | 10 | 10.0 | 10 |
| Bordetella pertussis | 1 (0.1) | 28 | 28.0 | 28 |
| Mycoplasma pneumoniae | 1 (0.1) | 17 | 17.0 | 17 |

Abbreviation: ARD=acute respiratory disease, RSV=respiratory syncytial virus.
