# Supplementary material for: Occurrence and disease burden of respiratory syncytial virus and other respiratory pathogens in adults aged ≥65 years in community: A prospective cohort study in Japan
Source: Influenza Other Respir Viruses. 2021 Nov 3;16(2):298–307. doi: 10.1111/irv.12928 (PMC8818832; doi:10.1111/irv.12928)
Supplement: Supplementary file 2 — Table S2: Summary of Symptom and Severity at ARD Diagnosis by Investigators (Pathogens Detected in >10 Samples) [file IRV-16-298-s002.docx]

Table 2_SuppInfo: Summary of Symptom and Severity at ARD Diagnosis by Investigators (Pathogens Detected in >10 Samples)

| **All ARD or ARD with pathogen detected** | **All** | **RSV** | **Influenza A/H1** | **Human Metapneumovirus** | **Human Rhinovirus/Enterovirus** | **Coronavirus OC43** | **Parainfluenza 3** |
| --- | --- | --- | --- | --- | --- | --- | --- |
|  | **N=459 n%** | **N=24 n%** | **N=11**  **n%** | **N=12**  **n%** | **N=105**  **n%** | **N=12**  **n%** | **N=20**  **n%** |
| **Upper respiratory symptoms** | | | | | | | |
| Nasal congestion | | | | | | | |
| None | 252 (54.9) | 9 (37.5) | 5 (45.5) | 7 (58.3) | 47 (44.8) | 4 (33.3) | 10 (50.0) |
| Mild | 177 (38.6) | 7 (29.2) | 6 (54.5) | 5 (41.7) | 51 (48.6) | 6 (50.0) | 10 (50.0) |
| Moderate | 27 (5.9) | 8 (33.3) | 0 | 0 | 6 (5.7) | 2 (16.7) | 0 |
| Severe | 3 (0.7) | 0 | 0 | 0 | 1 (1.0) | 0 | 0 |
| Sore throat | | | | | | | |
| None | 148 (32.2) | 6 (25.0) | 2 (18.2) | 3 (25.0) | 30 (28.6) | 4 (33.3) | 3 (15.0) |
| Mild | 240 (52.3) | 11 (45.8) | 7 (63.6) | 6 (50.0) | 55 (52.4) | 8 (66.7) | 12 (60.0) |
| Moderate | 68 (14.8) | 7 (29.2) | 2 (18.2) | 2 (16.7) | 19 (18.1) | 0 | 5 (25.0) |
| Severe | 3 (0.7) | 0 | 0 | 1 (8.3) | 1 (1.0) | 0 | 0 |
| **Lower respiratory symptoms** | | | | | | | |
| Cough | | | | | | | |
| None | 129 (28.1) | 5 (20.8) | 2 (18.2) | 1 (8.3) | 20 (19.0) | 3 (25.0) | 5 (25.0 |
| Mild | 234 (51.0) | 11 (45.8) | 6 (54.5) | 7 (58.3) | 55 (52.4) | 7 (58.3) | 7 (35.0) |
| Moderate | 88 (19.2) | 7 (29.2) | 3 (27.3) | 3 (25.0) | 27 (25.7) | 2 (16.7) | 8 (40.0) |
| Severe | 8 (1.7) | 1 (4.2) | 0 | 1 (8.3) | 3 (2.9) | 0 | 0 |
| Shortness of breath | | | | | | | |
| None | 413 (90.0) | 18 (75.0) | 9 (81.8) | 10 (83.3) | 98 (93.3) | 12 (100) | 17 (85.0) |
| Mild | 35 (7.6) | 4 (16.7) | 1 (9.1) | 2 (16.7) | 4 (3.8) | 0 | 1 (5.0) |
| Moderate | 11 (2.4) | 2 (8.3) | 1 (9.1) | 0 | 3 (2.9) | 0 | 2 (10.0) |
| Severe | 0 | 0 | 0 | 0 | 0 | 0 | 0 |
| Sputum Production | | | | | | | |
| None | 254 (55.3) | 9 (37.5) | 6 (54.5) | 2 (16.7) | 59 (56.2) | 8 (66.7) | 9 (45.0) |
| Mild | 166 (36.2) | 10 (41.7) | 4 (36.4) | 8 (66.7) | 34 (32.4) | 4 (33.3) | 9 (45.0) |
| Moderate | 32 (7.0) | 3 (12.5) | 1 (9.1) | 1 (8.3) | 10 (9.5) | 0 | 2 (10.0) |
| Severe | 7 (1.5) | 2 (8.3) | 0 | 1 (8.3) | 2 (1.9) | 0 | 0 |
| Wheezing | | | | | | | |
| None | 418 (91.1) | 18 (75.0) | 10 (90.9) | 10 (83.3) | 96 (91.4) | 12 (100) | 18 (90.0) |
| Mild | 32 (7.0) | 3 (12.5) | 1 (9.1) | 2 (16.7) | 8 (7.6) | 0 | 1 (5.0) |
| Moderate | 9 (2.0) | 3 (12.5) | 0 | 0 | 1 (1.0) | 0 | 1 (5.0) |
| Severe | 0 | 0 | 0 | 0 | 0 | 0 | 0 |
| **Systemic symptoms** | | | | | | | |
| Headache | | | | | | | |
| None | 352 (76.7) | 18 (75.0) | 7 (63.6) | 9 (75.0) | 85 (81.0) | 9 (75.0) | 15 (75.0) |
| Mild | 94 (20.5) | 5 (20.8) | 3 (27.3) | 3 (25.0) | 18 (17.1) | 3 (25.0) | 4 (20.0) |
| Moderate | 10 (2.2) | 1 (4.2) | 1 (9.1) | 0 | 2 (1.9) | 0 | 1 (5.0) |
| Severe | 3 (0.7) | 0 | 0 | 0 | 0 | 0 | 0 |
| Fatigue | | | | | | | |
| None | 286 (62.3) | 14 (58.3) | 7 (63.6) | 10 (83.3) | 71 (67.6) | 6 (50.0) | 13 (65.0) |
| Mild | 150 (32.7) | 8 (33.3) | 3 (27.3) | 2 (16.7) | 30 (28.6) | 6 (50.0) | 5 (25.0) |
| Moderate | 19 (4.1) | 1 (4.2) | 1 (9.1) | 0 | 4 (3.8) | 0 | 2 (10.0) |
| Severe | 4 (0.9) | 1 (4.2) | 0 | 0 | 0 | 0 | 0 |
| Fever | | | | | | | |
| None | 333(72.5) | 20(83.3) | 4(36.4) | 9 (75.0) | 70 (66.7) | 10 (83.3) | 11 (55.0) |
| Mild | 56 (12.2 | 2 (8.3) | 2(18.2) | 3 (25.0) | 14 (13.3) | 2 (16.7) | 6 (30.0) |
| Moderate | 11 (2.4) | 0 | 3(27.3) | 0 | 1 (1.0) | 0 | 0 |
| Severe | 0 | 0 | 0 | 0 | 0 | 0 | 0 |
| Feverishness | | | | | | | |
| None | 321 (69.9) | 16 (66.7) | 5 (45.5) | 8 (66.7) | 81 (77.1) | 6 (50.0) | 15 (75.0) |
| Mild | 121 (26.4) | 7 (29.2) | 3 (27.3) | 3 (25.0) | 21 (20.0) | 6 (50.0) | 5 (25.0) |
| Moderate | 12 (2.6) | 0 | 3 (27.3) | 0 | 3 (2.9) | 0 | 0 |
| Severe | 5 (1.1) | 1 (4.2) | 0 | 1 (8.3) | 0 | 0 | 0 |
| Myalgia | | | | | | | |
| None | 391 (85.2) | 22 (91.7) | 7 (63.6) | 10 (83.3) | 91 (86.7) | 12 (100) | 18 (90.0) |
| Mild | 51 (11.1) | 2 (8.3) | 2 (18.2) | 1 (8.3) | 13 (12.4) | 0 | 2 (10.0) |
| Moderate | 15 (3.3) | 0 | 2 (18.2) | 1 (8.3) | 1 (1.0) | 0 | 0 |
| Severe | 2 (0.4) | 0 | 0 | 0 | 0 | 0 | 0 |

Abbreviations: ARD=acute respiratory disease, N=total number of ARD episodes, n=number of ARD episodes in each category, RSV=respiratory syncytial virus.
