# Supplementary material for: Occurrence and disease burden of respiratory syncytial virus and other respiratory pathogens in adults aged ≥65 years in community: A prospective cohort study in Japan
Source: Influenza Other Respir Viruses. 2021 Nov 3;16(2):298–307. doi: 10.1111/irv.12928 (PMC8818832; doi:10.1111/irv.12928)
Supplement: Supplementary file 3 — Data S1. Supporting information [file IRV-16-298-s003.pdf]

Nasal congestion

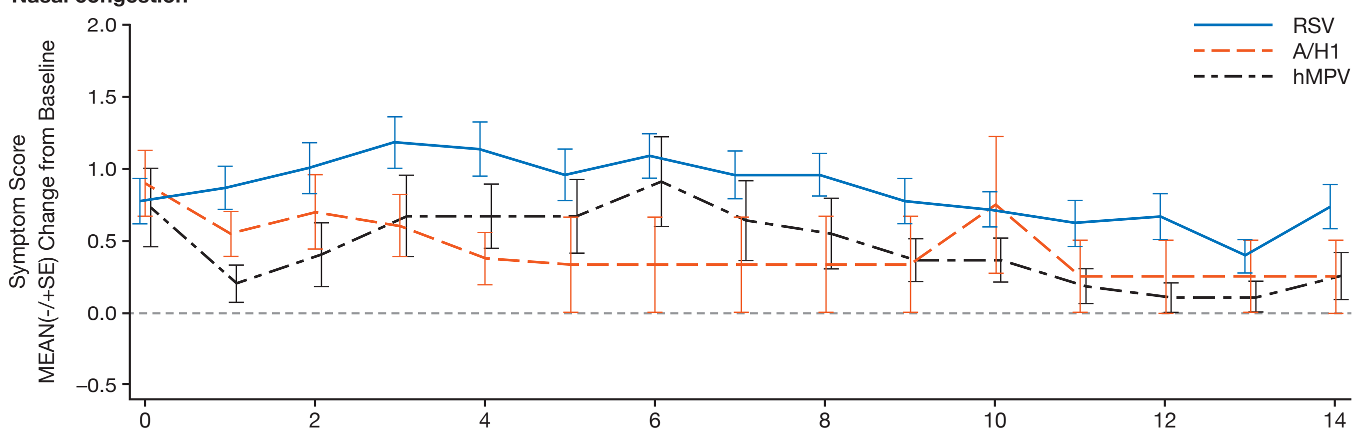

Number of participants

RSV	22	22	23	22	22	22	23	22	23	22	21	21	21	18	15
A/H1	10	11	10	10	8	6	6	6	6	6	4	4	4	4	4
hMPV	11	10	10	12	12	12	11	11	11	11	11	11	10	9	8

Sore throat

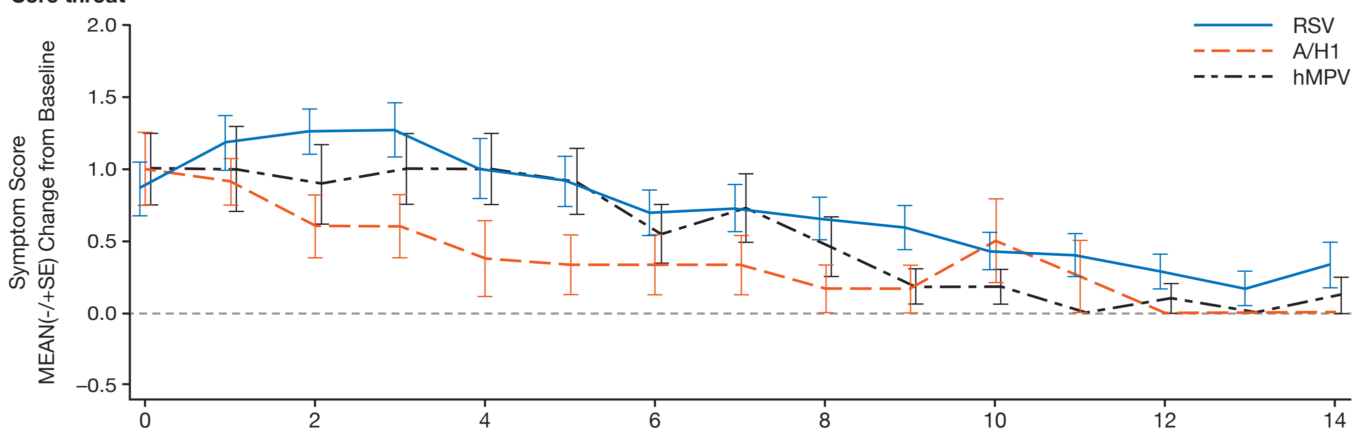

Number of participants

RSV	22	22	23	22	22	23	23	22	23	22	21	21	21	18	15
A/H1	10	11	10	10	8	6	6	6	6	6	4	4	4	4	4
hMPV	12	10	10	12	12	12	11	11	11	11	11	11	10	9	8

Cough

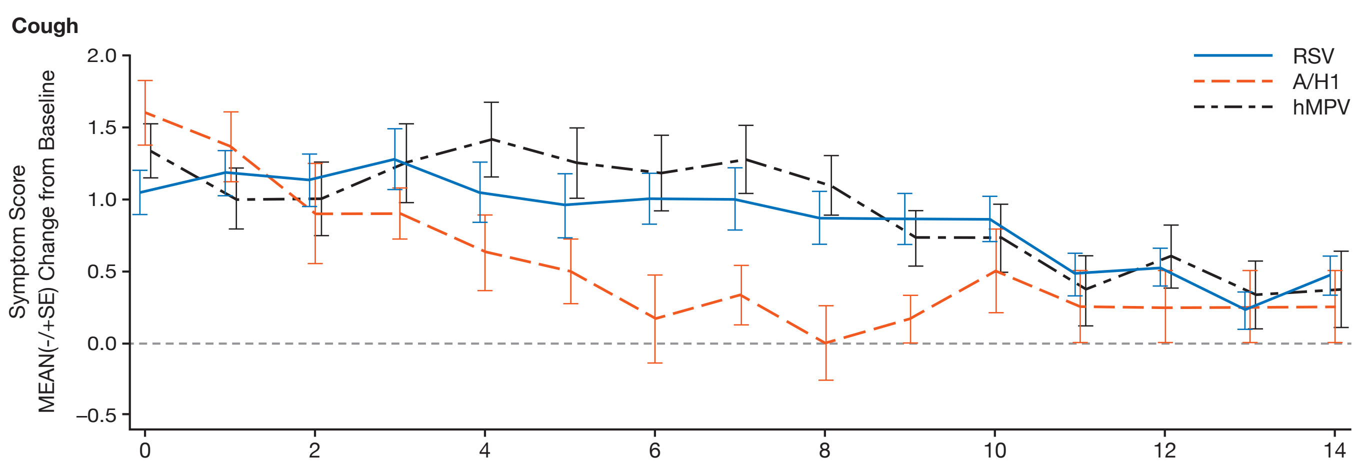

Number of participants

RSV	22	22	23	22	22	23	23	22	23	22	21	21	21	18	15
A/H1	10	11	10	10	8	6	6	6	6	6	4	4	4	4	4
hMPV	12	10	10	12	12	12	11	11	11	11	11	11	10	9	8

Short of breath

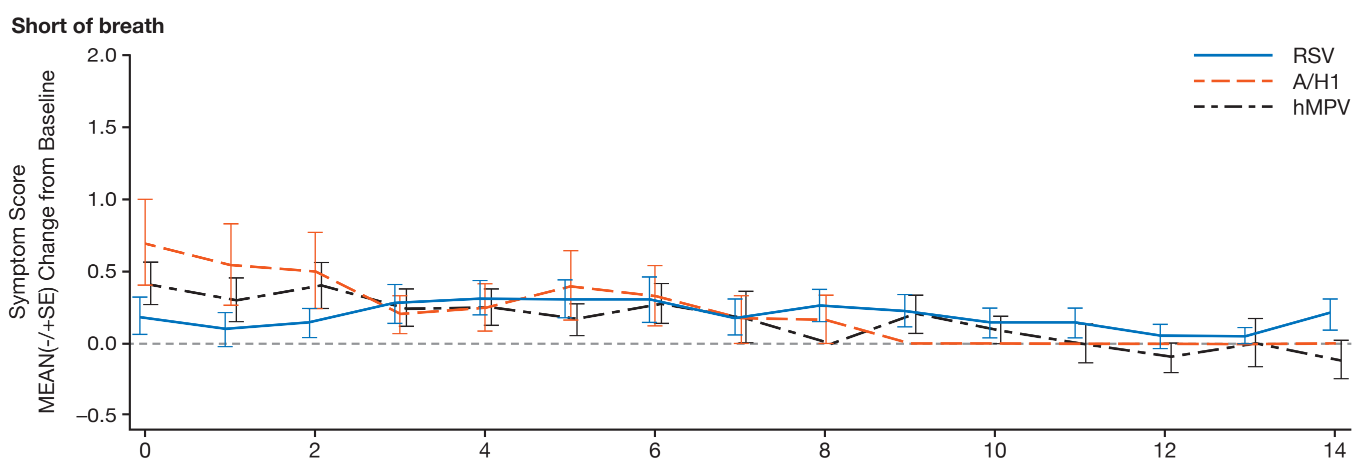

Number of participants

RSV	21	21	21	22	22	23	23	22	23	22	21	21	21	18	15
A/H1	10	11	10	10	8	5	6	6	6	6	4	3	4	4	4
hMPV	12	10	10	12	12	12	11	11	11	10	11	11	10	9	8

Expectoration

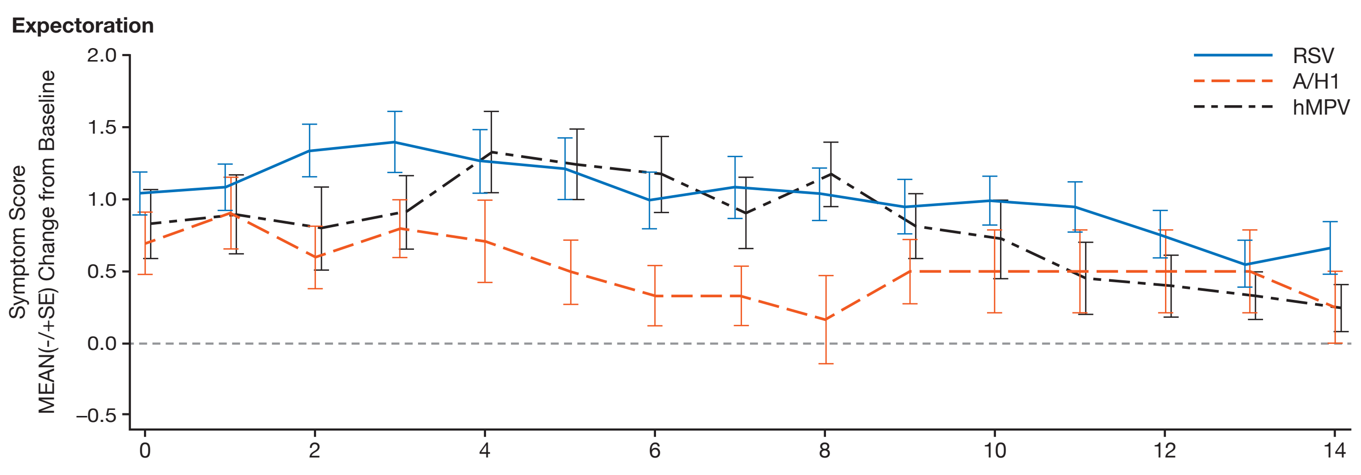

Number of participants

RSV	22	22	23	22	22	23	22	22	23	22	21	21	21	18	15
A/H1	10	11	10	10	7	6	6	6	6	6	4	4	4	4	4
hMPV	12	10	10	12	12	12	11	11	11	11	11	11	10	9	8

Wheezing

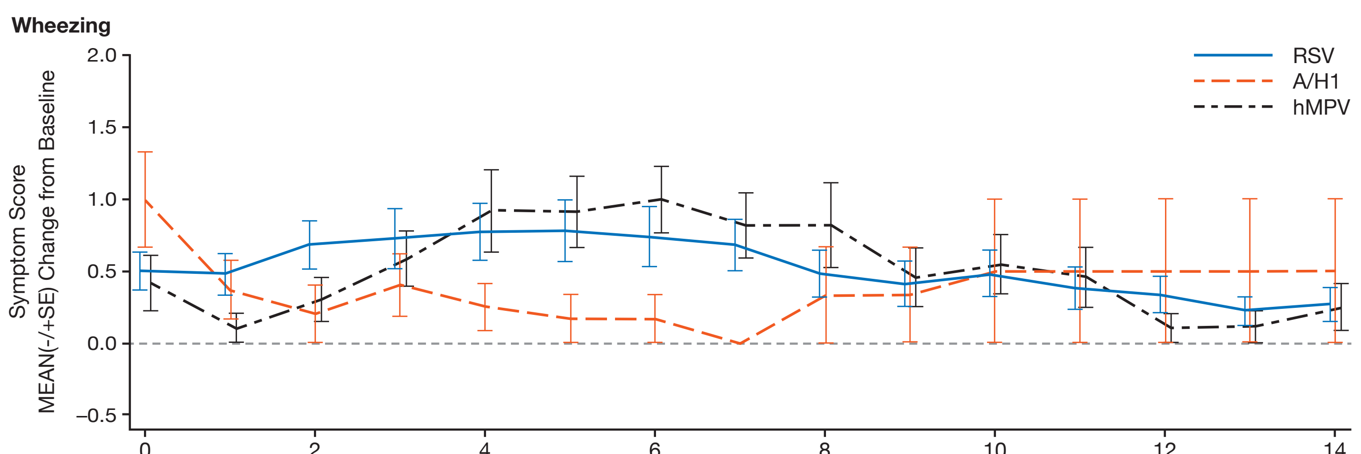

Number of participants

RSV	20	21	22	22	22	23	23	22	23	22	21	21	21	18	15
A/H1	9	11	10	10	8	6	6	6	6	6	4	4	4	4	4
hMPV	12	10	10	12	12	11	11	11	11	11	11	11	10	9	8

Headache

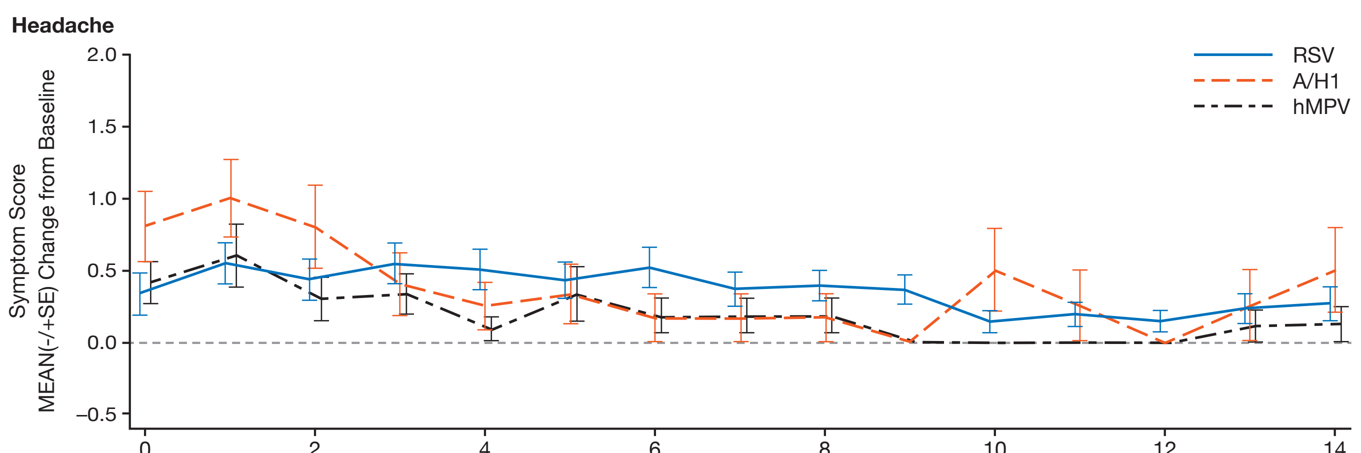

Number of participants

RSV	21	22	23	22	22	23	23	22	23	22	21	21	21	17	15
A/H1	10	11	10	10	8	6	6	6	6	6	4	4	4	4	4
hMPV	12	10	10	12	12	12	11	11	11	11	11	11	10	9	8

Fatigue

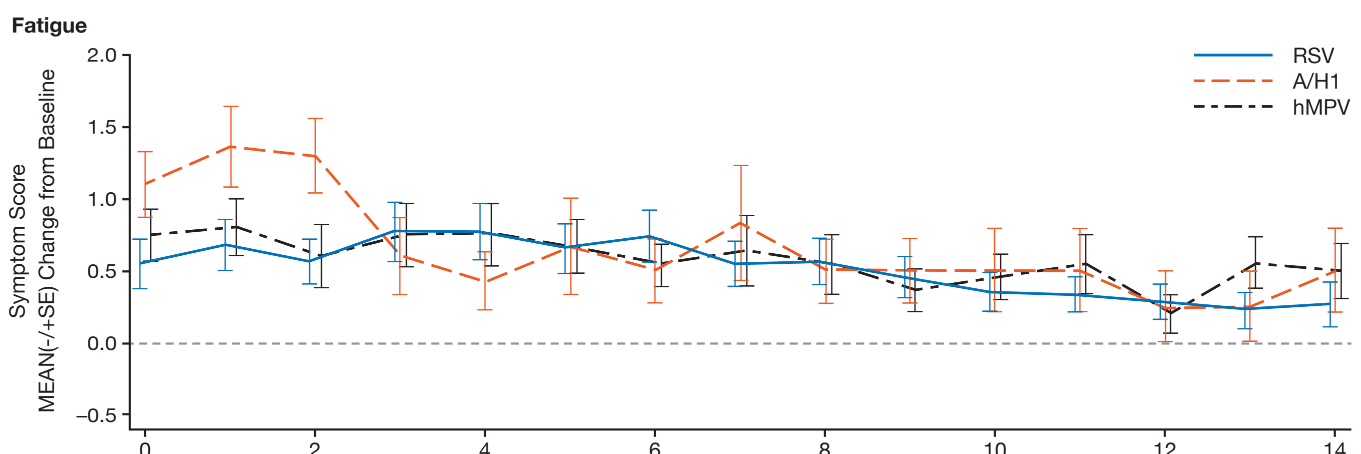

Number of participants

RSV	21	22	23	22	22	23	23	22	23	22	20	21	21	18	15
A/H1	10	11	10	10	7	6	6	6	6	6	4	4	4	4	4
hMPV	12	10	10	12	12	12	11	11	11	11	11	11	10	9	8

Feeling feverish

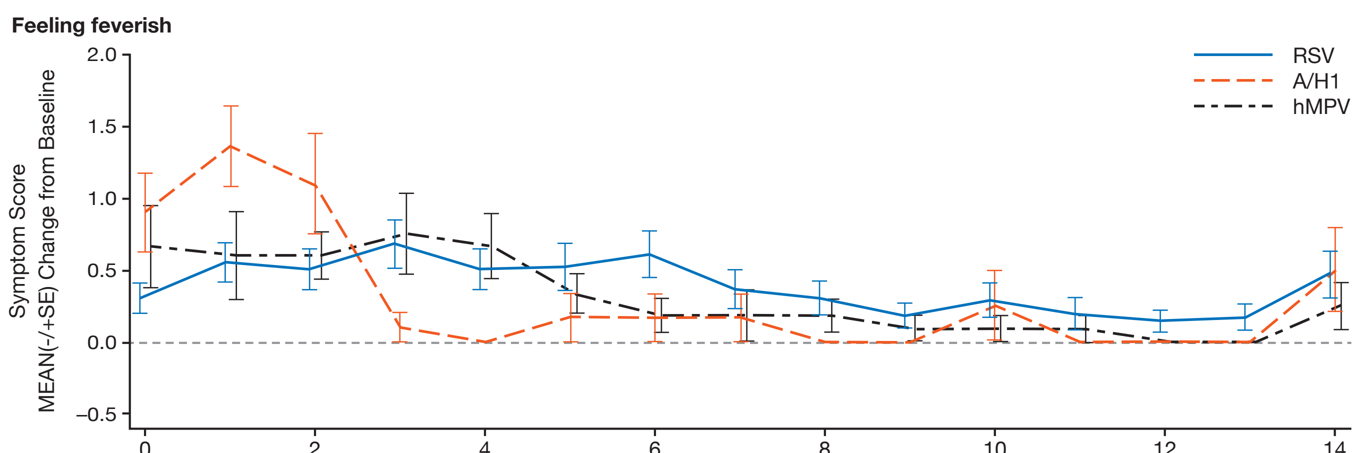

Number of participants

RSV	20	20	22	22	22	23	23	22	23	22	21	21	21	18	15
A/H1	10	11	10	10	8	6	6	6	6	6	4	4	4	4	4
hMPV	12	10	10	12	12	12	11	11	11	11	11	11	10	9	8

Myalgia

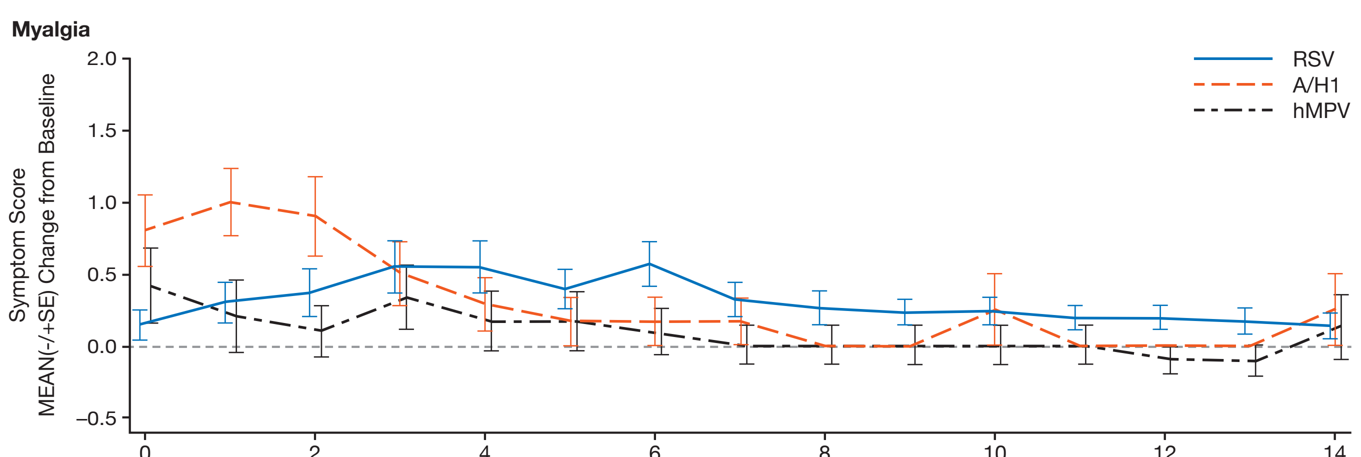

Number of participants

RSV	21	20	22	22	22	23	23	22	23	22	21	21	21	18	15
A/H1	10	11	10	10	7	6	6	6	6	6	4	4	4	4	4
hMPV	12	10	10	12	12	12	11	11	11	11	11	11	10	9	8

Neck pain

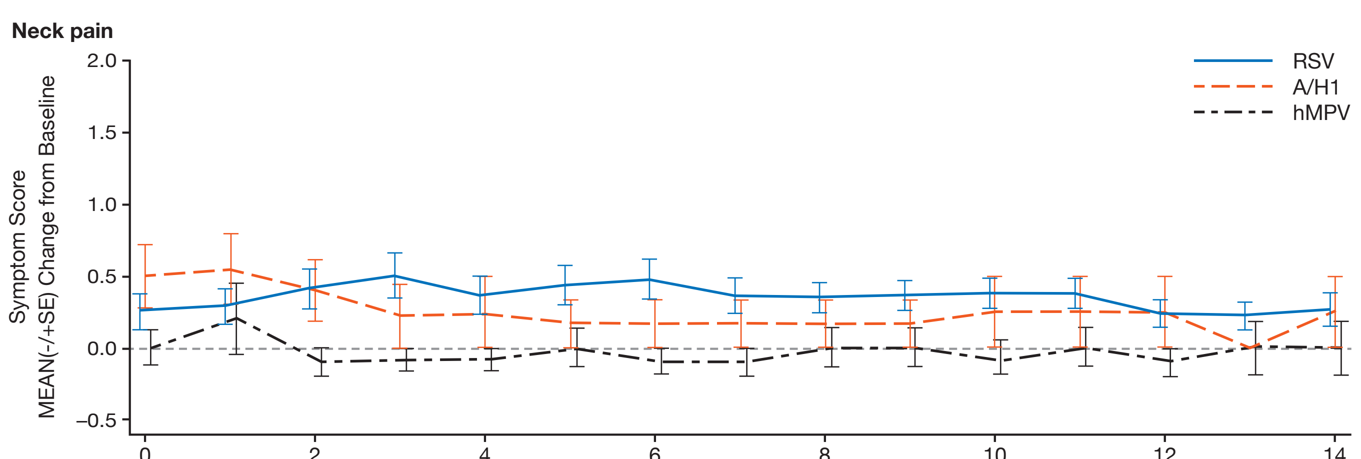

Number of participants

RSV	20	21	22	22	22	23	23	22	23	22	21	21	21	18	15
A/H1	10	11	10	9	8	6	6	6	6	6	4	4	4	4	4
hMPV	12	10	10	12	12	11	11	10	11	11	11	11	10	8	8

Interrupted sleep

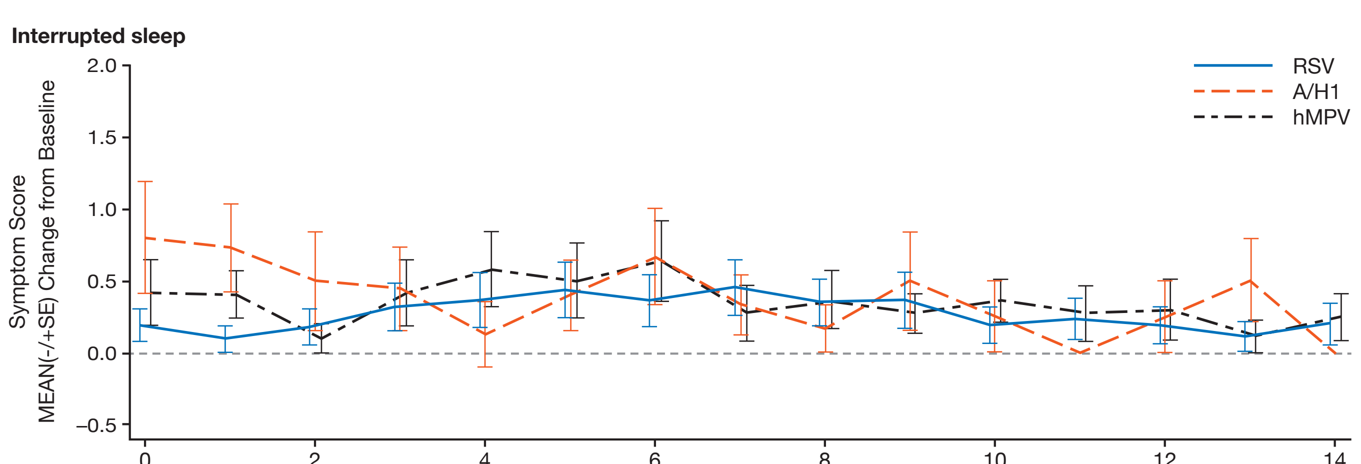

Number of participants

RSV	21	21	22	22	22	23	22	22	23	22	21	21	21	18	15
A/H1	10	11	10	9	8	5	6	6	6	6	4	4	4	4	4
hMPV	12	10	10	12	12	12	11	11	11	11	11	11	10	9	8

Loss of appetite

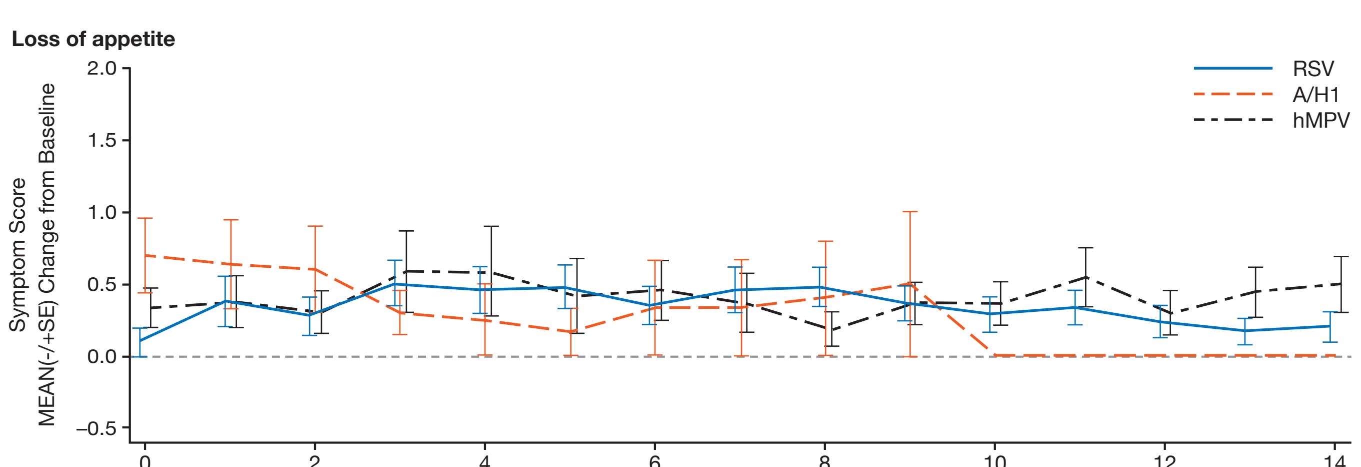

Number of participants

RSV	21	21	22	22	22	23	23	22	23	22	21	21	21	18	15
A/H1	10	11	10	10	8	6	6	6	5	6	4	3	4	4	4
hMPV	12	8	10	12	12	12	11	11	11	11	11	11	10	9	8
